# Is Allelopathic Activity of *Ipomoea murucoides* Induced by Xylophage Damage?

**DOI:** 10.1371/journal.pone.0143529

**Published:** 2015-12-01

**Authors:** Alejandro Flores-Palacios, Angélica María Corona-López, María Yolanda Rios, Berenice Aguilar-Guadarrama, Víctor Hugo Toledo-Hernández, Verónica Rodríguez-López, Susana Valencia-Díaz

**Affiliations:** 1 Centro de Investigación en Biodiversidad y Conservación, Universidad Autónoma del Estado de Morelos, Cuernavaca, Morelos, México; 2 Centro de Investigaciones Químicas, Universidad Autónoma del Estado de Morelos, Cuernavaca, Morelos, México; 3 Facultad de Farmacia. Universidad Autónoma del Estado de Morelos, Cuernavaca, Morelos, México; 4 Centro de Investigación en Biotecnología. Universidad Autónoma del Estado de Morelos., Cuernavaca, Morelos, México; University of New South Wales, AUSTRALIA

## Abstract

Herbivory activates the synthesis of allelochemicals that can mediate plant-plant interactions. There is an inverse relationship between the activity of xylophages and the abundance of epiphytes on *Ipomoea murucoides*. Xylophagy may modify the branch chemical constitution, which also affects the liberation of allelochemicals with defense and allelopathic properties. We evaluated the bark chemical content and the effect of extracts from branches subjected to treatments of exclusion, mechanical damage and the presence/absence of epiphytes, on the seed germination of the epiphyte *Tillandsia recurvata*. Principal component analysis showed that branches without any treatment separate from branches subjected to treatments; damaged and excluded branches had similar chemical content but we found no evidence to relate intentional damage with allelopathy; however 1-hexadecanol, a defense volatile compound correlated positively with principal component (PC) 1. The chemical constitution of branches subject to exclusion plus damage or plus epiphytes was similar among them. PC2 indicated that palmitic acid (allelopathic compound) and squalene, a triterpene that attracts herbivore enemies, correlated positively with the inhibition of seed germination of *T*. *recurvata*. Inhibition of seed germination of *T*. *recurvata* was mainly correlated with the increment of palmitic acid and this compound reached higher concentrations in excluded branches treatments. Then, it is likely that the allelopathic response of *I*. *murucoides* would increase to the damage (shade, load) that may be caused by a high load of epiphytes than to damage caused by the xylophages.

## Introduction

Herbivory is a selection pressure that releases defense mechanisms in plants, such as the synthesis of allelochemicals in response to damage [1]. As a response to herbivory, plants can modify their phenotype to increase the concentrations of constitutive compounds (*e*.*g*. lignin, tannins) or synthesize new compounds that act directly or indirectly on the herbivore [2, 3, 4, 5]. Moreover, certain allelochemicals, or combinations of these synthesized in response to herbivory have allelopathic properties that inhibit seed germination in their competitors [6, 7]. This increases the complexity of the biological interactions and could contribute to the explanation of patterns of assemblage of species [8, 9].

Trees provide resources for a wide variety of species, hosting and facilitating the interaction of different guilds (*e*.*g*. xylophages and epiphytic plants) [10, 11]. The chemical constituents of the host species influence the organisms they support [12, 13, 14] and may vary intra-specifically and as a function of herbivory [1]. It is known that certain xylophages (Coleoptera: Cerambycidae) prefer species that are chemically similar [12] and that concentrations of tannins increase in the affected area of plants damaged by curculionid xylophages [15]. It is kwon also that bark of trees and epiphytic lichens may exert an allelopathic effect on epiphytes [16, 17]. For example, the seed germination of the epiphyte orchid *Dendrobium aphyllum* (Roxb.) C.E.C. Fisch is reduced by bark extracts of those trees in which the species rarely occur [17], and the host *Ipomoea murucoides* Roem. & Schult (Convolvulaceae) has an allelopathic effect on seed germination in the epiphytic bromeliad *Tillandsia recurvata* (L) L. [14].

There is evidence of a negative association between the presence of xylophages and species of epiphytic bromeliads on different host species [18]. This is most clearly seen in the *Ipomoea murucoides*-*Tillandsia recurvata* interaction, where this epiphyte is not abundant but the number and activity of xylophages is greater than in other host species [18]. The guild of xylophages is characterized by boring holes in their host tree while feeding on the conductive tissues [19]. It is likely that when the xylophages bore tunnels they promote the liberation of allelochemicals that act as allelopathics on the germination of *T*. *recurvata* seeds. It is also known that epiphytes can mechanically weaken their hosts [20] and it is likely that by themselves they trigger the synthesis of allelopathics in the hosts. The objective of this study was to determine the effect of intentional damage (simulating damage by xylophages) in branches of *I*. *murucoides* on the germination of *T*. *recurvata* seeds and distinguish this from the effect of epiphytes on the host. It is hypothesized that the activity of xylophages on the host, simulated by the intentional damage, induces the liberation of allelochemicals with allelopathic properties on the germination of seeds of this epiphyte.

## Materials and Methods

### Species and study area

The study area was located in San Andrés de la Cal, in Tepoztlan, in Morelos state, Central Mexico (18°57’22.2”W, 99°06’50.2”N, 1495 m asl). This area belongs to the people of San Andrés de la Cal, they are represented by the President of communal lands who gave us permission to carry out this survey. Mean annual precipitation is 1098 mm and mean temperature ranges from 12°C to 18°C [21]. The vegetation is tropical dry forest, characterized by an open canopy, with trees < 16 m height [22]. At the study area, the dominant tree species include *Sapium macrocarpum* Müll. Arg. (Euphorbiaceae), *Bursera copallifera* (DC.) Bullock, *B*. *fagaroides* (Kunth) Engl., *B*. *glabrifolia* (Kunth) Engl. (Burseraceae), *Ipomoea murucoides* (Convolvulaceae), *Conzattia multiflora* (B.L. Rob.) Standl. and *Lysiloma acapulcense* (Kunth) Benth (Fabaceae) [23].

*Ipomoea murucoides* is a deciduous tree that flowers from October to March and reaches approximately 8 m in height in the tropical dry forests of central and southern Mexico [24]. It has been reported that plants of this genus (*e*.*g*. *I*. *tricolor*) produce allelopathically active alkaloids and resin glycosides [25, 26]. In the case of *I*. *murucoides*, it contains allelopathics that inhibit the germination of the epiphyte *T*. *recurvata* through dichloromethane bark extract [14, 27]. On the other hand, it is known that extracts of diverse organs from different *Ipomoea* species have insecticidal effects on herbivores [28, 29].

At the study site, *Tillandsia recurvata* comprises 72% of the epiphytic individuals and is mainly distributed on hosts from the family Burseraceae, such as *Bursera bipinnata* (DC.) Engl., *B*. *copallifera* and *B*. *glabrifolia*. However, in others such as *B*. *fagaroides*, *I*. *murucoides* and *I*. *pauciflora*, this epiphyte is practically absent [23]. It has been reported that these host species may harbor 63.6% of the xylophagous insects present in the study area; these insects were mainly Coleoptera (41.8%) or those that cause boring damage to the bark such as Hymenoptera (Formicidae) [18]. Within the order Coleoptera, the family Cerambycidae was the most represented (67.4% of coleopterans), followed by Tenebrionidae (19.6%), Buprestidae (4.4%), Bostrichidae and Elateridae (both 2.2%) [18]. We state that the field studies did not involve endangered or protected species

### Field experimentation and processing of plant material

In Nov. 2012, along a 1 km path in the “Cerro de la Cruz”, in San Andrés de la Cal, ten individuals of *I*. *murucoides* (height > 4m; mean DBH at 1.3 m = 21.7 cm ± 5.1 cm) were randomly selected. Using a 6 m ladder, eight branches were chosen at random from the exterior stratum of the tree (hereafter, all values are mean ± 1 SE, unless otherwise stated, diameter = 1.7 cm ± 0.05 cm, and length = 1.2 m ± 0.5 m). The branches chosen all appeared healthy (with leaves or flowers, no dry areas, and green bark) and had no epiphytes or apparent damage in the bark.

In order to determine whether the association between the xylophages and epiphytes on *I*. *murucoides* is mediated by allelochemicals liberated when the xylophages pierce the branches of the host, each treatment was assigned at random to each of the eight selected branches on each trees of *I*. *murucoides*. The factors combined to obtain the eight branch treatments were: exclusion, intentional mechanical damage and the presence of *T*. *recurvata*. Exclusion (excluded *vs*. non-excluded branches) consisted of covering the branch with a mesh bag in order to avoid oviposition and decrease the activity of xylophages over the course of the experiment. Intentional damage to the branches (damaged *vs*. non-damaged branches) attempted to mechanically simulate the damage caused by xylophages and thereby weaken the branches in order to promote a higher xylophage activity. The intentional damage consisted of making 2–3 mm wide groove, lifting the bark along 25% of the branch length. The groove was made with the hook of a diametric measuring tape (Forestry Suppliers Metric Fabric Diameter Tape). The third factor was the presence/absence of *T*. *recurvata*. This was to determine whether epiphytes could independently activate allelopathic mechanisms in the host. In the treatment of branches with epiphytes, four individuals of *T*. *recurvata*, collected from hosts of the same 1 km transect, were transplanted. The bromeliads were adults of more than 10 cm in diameter and were fixed at equal distances apart on the branch with plastic straps. Combination of these factors produced the following eight treatments: 1) excluded branches, with *T*. *recurvata* and with damage, 2) excluded branches, with *T*. *recurvat*a and without damage, 3) excluded branches, without *T*. *recurvata* and with damage, 4) excluded branches, without *T*. *recurvata* and without damage, 5) non-excluded branches, with *T*. *recurvata* and with damage, 6) non-excluded branches, with *T*. *recurvata* and without damage, 7) non-excluded branches, without *T*. r*ecurvata* and without damage, and finally 8) non-excluded branches, without *T*. *recurvata* and without damage.

In order to avoid leaching of allelochemicals due to the rainy season (Oct-May), the branches were cut from the trees in May 2013 and transported to the ecology laboratory of Centro de Investigación en Biodiversidad y Conservación-Universidad Autónoma del Estado de Morelos (CIByC-UAEM) for processing. The branches were cleaned of all external agents (*e*.*g*. lichens) and before stripping the bark, the following data were recorded: number of orifices evidently caused by xylophages (hereafter orifices), total branch length (cm, length of main branch plus length of secondary branches), diameter of main branch base (mm) and non-intentional damage (cm) (*i*.*e*. pooled length measurements of bark scars, girdled branches or orifices). The percentage of non-intentional damage was obtained by dividing the non-intentional damage value by the total length of the branch and multiplying by 100.

Also recorded were the number and identity of the larvae and imagoes of insect xylophages found in the branches of the eight treatments. These were preserved in 60% ethanol and identified to taxonomic group or, when possible, to species. Larval identification was conducted at the Insect Collection of the Universidad de Morelos (CIUM) at the CIByC-UAEM. Keys used for identification were those used by Costa and collaborators [30] and Chu [31].

The bark was removed from the branches and dried in a fan oven (FD 115-UL, Binder) at 30°C until reaching a constant dry weight. The dry bark was ground to < 3 mm in an electric mill (PULVEX S. A. de C.V. model Mini-100). Disregarding the identity of the tree, the resulting dry bark powder samples were pooled according to branch treatment and stored in darkness at -15° C until use. The dry bark material from the eight treatments was subjected to extraction by maceration at room temperature with dichloromethane. The dichloromethane extract from *I*. *murucoides* was chosen because it has been shown to produce the highest inhibition (58%) of seed germination in *T*. *recurvata* [14, 27]. Following the protocol used by Valencia-Díaz and collaborators [14] to obtain dichloromethane extracts, fats were first removed from the sample by maceration with hexane (three periods of 72 h each) at ambient temperature and then the dichloromethane extracts were obtained by repeating the protocol used with the hexane. Hexane and dichloromethane solubilized compounds that are water insoluble or that have low water solubility (i.e. terpenoids) [32]. Following filtration, extracts were concentrated under vacuum (Rota-evaporator Buchi R-200) at 39°C for hexane and dichloromethane. Dry extracts were stored at -15°C until use; however, only the dichloromethane extracts were used in the germination experiments.

### Seed collection and germination tests

The germination experiments were carried out with *T*. *recurvata* seeds collected in April 2013. We collected *T*. *recurvata* seed capsules from 18 individual plants. Seeds were removed from the capsules once dehiscence had taken place. We removed the coma hairs from the seeds to facilitate manipulation and reduce the risk of contamination during the experiments. *Tillandsia recurvata* seeds were joined in a seed batch; then they were randomized and sterilized prior to sowing using 0.25% sodium hypochlorite solution, with subsequent rinsing three to four times in sterile distilled water to completely remove remnants of the sodium hypochlorite.

We hypothesized that extracts obtained from the artificially damaged branches of *I*. *murucoides* would have allelopathic properties on *T*. *recurvata* seed germination. To test this hypothesis, we applied dichloromethane extract obtained from each of the eight treatments at its most inhibitory concentration [1 μg/mL of distilled water; 14]. Ten seeds of *T*. *recurvata* were placed in a Petri dish using filter paper as substrate. Twenty-four Petri dishes were used for each treatment along with four Petri dishes with distilled water but without extract (control). In order to exclude the effects of factors other than allelopathy (e.g. non-viable seeds or dormancy) from the calculation, percentage of inhibition of seed germination was calculated relative to that recorded in the control. Percentage inhibition of seed germination was therefore calculated according to the following equation:

$$\frac{{\bar{X}}_{0}-X_{i}}{n}\times 100$$

Where, ${\bar{X}}_{0}$ is the mean number of germinated seeds in the control, *X*_*i*_ is the number of germinated seeds under a specific concentration and *n* is the number of seeds (n = 10) in the Petri dish. Positive and negative values indicate inhibition and promotion of germination, respectively, while zero indicates a complete absence of bark host effect.

As with the dichloromethane extracts, dimethyl sulfoxide (DMSO: (CH3)2SO, Fermont) was used, at a maximum concentration of 1% (v/v), in order to prepare the solutions [14]. In all germination tests, petri dishes were placed within an environmental growth chamber (Scorpion Scientific A 50624, Mexico) for 10 days under a photoperiod regime of 12 h light/ 12 h dark and a constant temperature of 30°C. Since bromeliad seeds do not produce a radicle during the early germination process, seeds were considered to have germinated upon rupture of the testa [33].

## Chemical analysis

In order to know the chemical constituents among extracts obtained from the branch treatments 21 mg of each of the eight samples (one sample per branch treatment), were solubilized in ethyl acetate (1 mL). Solid phase extraction was done using 1 mL cartridges SUPELCO® with a normal phase (LC-SI). 1 mL of ethyl acetate was recovered from the sample loaded in the cartridge. Just 400 μL were evaporated. Six mL of ethyl acetate was recovered from washing cartridge. Solvent was evaporated. The eight samples were reconstituted in dichloromethane (1 mL) and injected in GC-MS. Aliquots (150 μL) from these solutions were injected into a gas chromatograph (Agilent 6890) coupled to a mass detector [Agilent 5379 series N, 30–550 atomic mass units, electron impact ionization (70 eV)], equipped with an HP5-MS capillary column (25 m × 0.20 mm × 0.33 M film thickness, 5% phenylmethyl siloxane stationary phase). Helium was the carrier gas at a flow rate of 1 mL min^−1^. All extracts were analyzed using the following temperature program: 1–45°C 1 min, with an increase of 10°C min^−1^ until reaching 250°C and then 260°C was maintained for 30 min. The database used for comparison of the results was NIST-MS version 1.7a.

## Data analyses

The variables total length of branch, diameter, percentage of non-intentional damage, number of orifices and number of xylophages were analyzed using an analysis of variance with three factors [34], where the main factors and their levels were: exclusion (excluded and non-excluded branches), intentional damage (damaged and undamaged branches) and epiphytes (presence and absence). Differences among factors and interactions were analyzed with the Tukey multiple comparisons test [34]. Due to the nature of the data, and to comply with the assumptions of normality and homogeneity of variance, the variable percentage of non-intentional damage was transformed using an arcsin function, and the variables number of orifices and number of xylophages were square root transformed [34].

The percentage of inhibition of germination of *T*. *recurvata* seeds caused by the dichloromethane extracts was analyzed with an analysis of variance of three factors with two levels per factor. To normalize the data and make the variances uniform, the data were transformed using the arcsin function [34]. The factors were exclusion (excluded and non-excluded branches), intentional damage (branches with and without damage) and epiphyte presence/absence. Differences among factors and interactions were analyzed with the Tukey multiple comparisons test [34]. All analysis was performed using the program Stata 13 [35].

We hypothesized that bark extracts would form associations according to their chemical constituents and that those extracts obtained from branches under intentional damage would have allelopathic properties on *T*. *recurvata* seed germination. In order to test this hypothesis we performed a principal components analysis (PCA) [36] using as variables the chemical compound concentration (%) within the extracts of each treatment and the mean percentage of inhibition of seed germination of *T*. *recurvata* exerted by these extracts. PCA analysis was based on the correlation matrix, for each eigenvector (principal component), we considered as significant, those variables that contribute with at least 10% to the eigenvector normalized length and that at the same time were highly correlated with it (r ≥ 0.6) [36].

## Results

In three of the ten trees in which the experiment was conducted, mesh bags used to achieve exclusion were lost, for this reason only the data derived from the branches (n = 56) of seven trees were considered. General measurements of branches were: length of 188.62 cm ± 13.60 cm, diameter of 17.24 mm ± 0.53 mm, 0.82 ± 0.23 orifices, 1.28 ± 0.30 xylophages and non-intentional damage of 8.30 cm ± 1.21 cm. There were no significant differences of length of branches, diameter, number of xylophages, number of orifices and natural damage between levels of the main factors (Table 1). Furthermore, the treatment interactions did not reveal statistical differences for any measured variable (all *F*_*1*,*48*_ < 2.3, all *P* > 0.05).

**Table 1:** Mean (± SE) of the variables measured in branches of the tree *Ipomoea murucoides* subjected to three main treatments (factors): exclusion (excluded *vs*. non-excluded branches), intentional damage (branches with intentional damage *vs*. branches without intentional damage) and with the presence/absence of *T*. *recurvata* (N = 28). No single variable showed statistical differences between factor levels (P > 0.05). F _1,48_ values of the variables within each factor were in italics and in parenthesis.

Treatments	Total length (cm)	Diameter (mm)	No. of xylophages	No. of orifices	Natural damage (%)
Non-excluded	207.92 ± 24.14 (*2*.*13*)	17.72 ± 0.88 (*0*.*77*)	1.46 ± 0.40 (*0*.*34*)	0.71 ± 0.30 (*0*.*36*)	7.96 ± 2.22 (*1*.*97*)
Excluded	169.33 ± 12.00	16.77 ± 0.60	1.07 ± 0.44	0.93 ± 0.36	8.64 ± 1.02
Without intentional damage	188.74 ± 16.12 (*0*.*0001*)	16.93 ± 0.57 (*0*.*34*)	1.32 ± 0.36 (*0*.*01*)	1.03 ± 0.43 (*0*.*42*)	9.21 ± 1.20 (*0*.*26*)
With intentional damage	188.51 ± 22.23	17.57 ± 0.91	1.25 ± 0.47	0.6 ± 0.19	7.40 ± 1.39
Without epiphytes	173.07 ± 18.90 (*1*.*38*)	16.92 ± 0.83 (*0*.*36*)	1.07 ± 0.44 (*0*.*5*)	0.82 ±0.37 (*0*.*01*)	10.98 ± 2.12 (*3*.*72*)
With epiphytes	204.18 ± 19.50	17.58 ± 0.67	1.50 ± 0.40	0.82 ± 0.29	5.63 ± 0.96

A total of 85 arthropods were found, of which 83 were larvae and two were adults one belonged to *Brachymyrmex* sp. (Hymenoptera: Formicidae) and the other was a bark-bettle (Curculionidae: Scolytinae). Due to the fact that the majority of individuals found were larvae, identification was limited. Of the total number of arthropods, the majority (83.52%) belonged to the order Coleoptera, followed by Lepidoptera (12.94%), Hemiptera (1.17%), Hymenoptera (1.17%) and by the class Arachnida (1.17%). Four families of the order Coleoptera were identified, with Cerambicidae accounting for most individuals (92.95%), followed by Buprestidae (4.22%), Curculionidae (1.40%) and Tenebrionidae (1.40%). The analysis only included the insect xylophages; in this case, Coleoptera.

### Germination tests

The percentage of inhibition of *T*. *recurvata* seeds was different in the exclusion treatments (*F*_1,184_ = 86.30, P < 0.001), with lower germination recorded on application of the extract derived from the excluded (51.77% ± 1.68% inhibition) than non-excluded (30.31% ± 1.59% inhibition; Tukey < 0.05) branches. There were no differences (*F*_*1*,*184*_ = 1.60, *P* = 0.20) in the inhibition of *T*. *recurvata* seed germination on application of extract derived from branches with intentional damage (39.58% ± 1.98% inhibition) and from those without intentional damage (42.50% ± 1.95% inhibition; Tukey > 0.05). The percentage of inhibition of *T*. *recurvata* seed germination was equal (*F*_*1*,*184*_ = 0.60, *P* = 0.43) when tested with extracts derived from branches with (40.31% ± 2.09%) and without (41.80% ± 1.83%; Tukey > 0.05) epiphytes.

Regarding the interaction between each pair of main factors, the *T*. *recurvata* seeds were equally inhibited by the dichloromethane extract obtained from excluded branches and from those with damage (*F*_*1*,*184*_ = 1. 47, *P* = 0.0006; Table 2). There was an effect of the interaction between exclusion and presence/absence of epiphytes (*F*_*1*,*184*_ = 5.03, *P* = 0.02) on the germination of *T*. *recurvata* seeds, with greater inhibition found in the seeds treated with extracts derived from excluded branches, regardless of the presence/absence of epiphytes (Tukey < 0.05; Table 2). The interaction between intentional damage and the presence/absence of epiphytes also affected the percentage of inhibition of germination in *T*. *recurvata* seeds (*F*_*1*,*184*_ = 11.9, *P* = 0.0006); however, multiple comparisons testing did not establish significant differences between the percentages of inhibition of germination, for which reason this result may be considered spurious. None of the interactions among the three main factors on inhibition of *T*. *recurvata* seed germination were statistically significant (*F*_*1*,*184*_ = 0.26, *P* = 0.61; Table 3).

**Table 2:** Mean (± SE) of the percentage of inhibition of the germination of *T*. *recurvata* seeds in response to the application of dichloromethane extracts obtained from branches of *I*. *murucoides*. The data refer the interaction between pairs of the main factors of the experiment: exclusion (excluded *vs*. non-excluded branches), intentional damage (branches with intentional damage *vs*. branches without intentional damage) and with the presence/absence of *T*. *recurvata* (N = 48). Within each section of the table, different letters indicate statistical differences between the interaction of the pairs of treatments (Tukey test P < 0.05).

Interaction between pairs of main factors	% Inhibition of seed germination
Non.excluded * without intentional damage	30.41 ± 14.14^a^
Non-excluded * with intentional damage	30.21 ± 17.07^a^
Excluded * without intentional damage	54.58 ± 15.6^a^
Excluded * with intentional damage	48.96 ± 17.04^a^
Non-excluded * without epiphytes	33.54 ± 14.66^b^
Non-excuded * with epiphytes	27.08 ± 16.00^b^
Excluded * without epiphytes	50.00 ± 17.38^a^
Excluded * with epiphytes	53.54 ± 15.50^a^
Without intentional damage * without epiphytes	39.37 ± 17.80^a^
Without intentional damage * with epiphytes	45.62 ± 20.10^a^
With intentional damage * without epiphytes	44.16 ± 18.10^a^
With intentional damage * with epiphytes	35.00 ± 19.79^a^

**Table 3:** Percentage of inhibition of the germination (mean ± SE) of *T*. *recurvata* seeds in response to the application of dichloromethane extracts obtained from branches of *I*. *murucoides*. The data shown correspond to the triple interaction of the three main factors of the experiment: exclusion (excluded *vs*. non-excluded branches), intentional damage (branches with intentional damage *vs*. branches without intentional damage) and with the presence/absence of *T*. *recurvata* (N = 24).

Exclusion	Intentional damage	Epiphytes	% Inhibition of seed germination
Non-excluded	Without	Without	30.41 ± 12.67
Non-excluded	Without	With	30.41 ± 15.73
Non-excluded	With	Without	36.66 ± 16.06
Non-excluded	With	With	23.75 ± 15.83
Excluded	Without	Without	48.33 ± 17.85
Excluded	Without	With	60.83 ± 9.74
Excluded	With	Without	51.66 ± 17.11
Excluded	With	With	46.25 ± 16.90

### Chemical content

**Fig 1:**
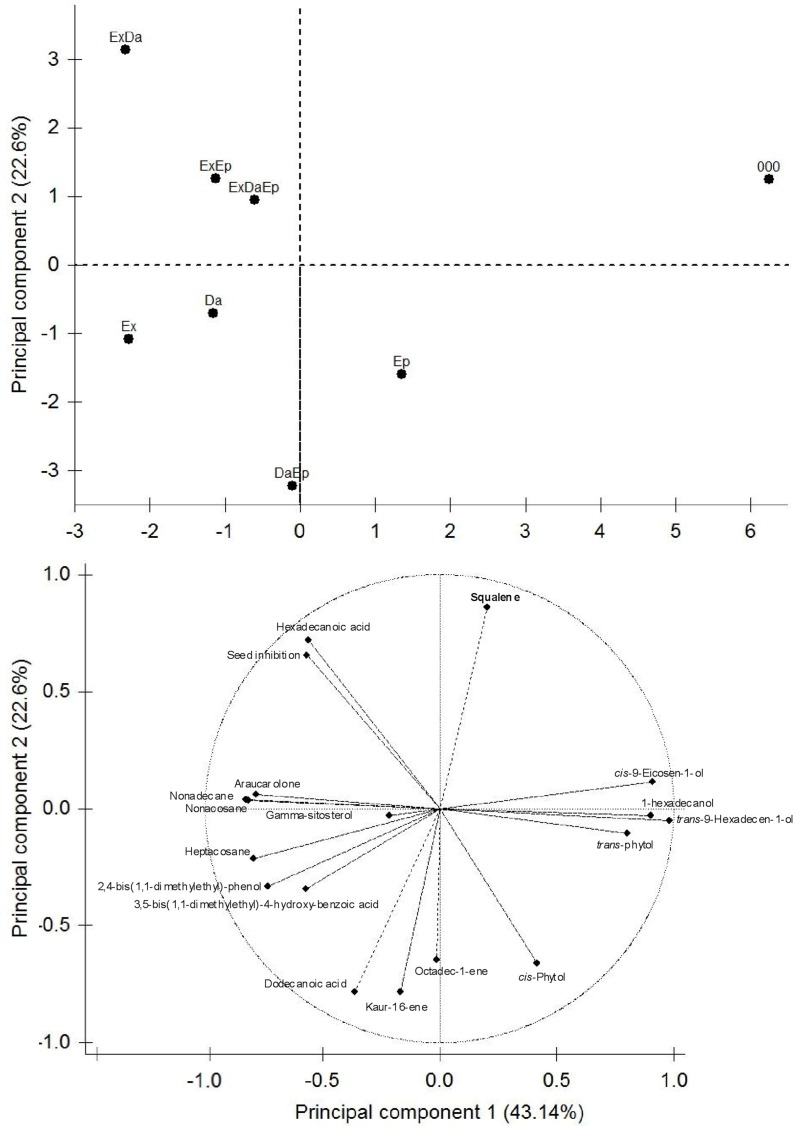
Principal components based on the chemical compounds found in dichloromethane extracts from eight branch treatments (see methods) also is shown the correlation projections of each chemical compound. Branches excluded (Ex), damaged branches (Da), Presence of epiphytes (Ep), control means those branches without any treatment (000).

GC-MS of the eight extracts identified 17 different chemical compounds (Table 4, Fig 1), one of them, araucarolone, reported here for second time [37]. The first two principal components (PC) explained 65.7% of the variance. PC1 explained 43.1% of the variance, within this component three chemical compounds contributed with 33.6% of the total length of the eigenvector and were highly correlated with it (r ≥ |0.9|) (Table 4). PC2 explained 22.6% of the variance, and in this component five chemical compound along with inhibition of seed germination of *T*. *recurvata* (r ≥ |0.65|) contributed with 82% of the total length of the eigenvector (Table 4). Based on their chemical composition, first component separated extracts obtained from branches subject to exclusion or damage respect to control branches; while branches whose treatments were to have only epiphytes were in the middle of the previous groups (Fig 1). Second component separated extracts obtained from branches with a unique treatment (excluded or damaged or with epiphytes, at bottom of the y-axis in Fig 1) and extracts obtained from branches without exclusion but subject to damage and epiphytes, respect to the control and excluded branches with damage and/or epiphytes (Fig 1).

**Table 4:** Retention time, coefficient of correlation and percentage of contribution of each variable to the total length of the PC1 and PC2. The analyzed variables were the inhibition of seed germination of *T*. *recurvata* and 17 chemical compounds identified with GC-MS in the bark of *Ipomoea murucoides*. In bold those chemical compounds whose percentage with contribution ≥ 10% to the eigenvalue length.

Retention time (min)	Chemical compund	PC1		PC2	
		r	Contribution (%)	r	Contribution (%)
14.73	2,4-bis(1,1-dimethylethyl)-phenol	-0.7	6.9	-0.3	2.7
**15.28**	**Dodecanoic acid**	-0.3	1.7	**-0.8**	**15**
16.91	3,5-bis(1,1-dimethylethyl)-4-hydroxy-benzoic acid	-0.6	4.2	-0.3	2.9
**18.41**	***cis*-phytol**	0.4	2.2	**-0.7**	**10.7**
**18.85**	**1-hexadecanol**	**0.9**	**10.5**	-0.1	0.0
**19.65**	**Hexadecanoic acid**	-0.6	4	**0.7**	**12.7**
**20.77**	**Kaur-16-ene**	-0.1	0.4	**-0.8**	**15.1**
**20.83**	***trans*-9-Hexadecen-1-ol,**	**0.9**	**12.4**	-0.1	0.1
21.12	trans-phytol	0.8	8.3	-0.1	0.3
**22.5**	***cis*-9-Eicosen-1-ol**	**0.9**	**10.7**	0.1	0.3
22.7	Octadec-1-ene	-0.1	0.0	-0.6	10.2
25.18	Nonadecane	-0.8	8.8	0.1	0.0
28.54	Heptacosane	-0.8	8.1	-0.2	1.1
**31.13**	**Squalene**	0.2	0.5	**0.9**	**18.1**
32.8	Nonacosane	-0.81	8.6	0.1	0.0
33.56	Araucarolone	-0.78	7.9	0.1	0.1
49.77	Gamma-sitosterol	-0.2	0.7	-0.0	0.1
	**Inhibition**	-0.6	4.1	**0.7**	**10.6**

## Discussion

Previous evidence shows that dichloromethane bark extract of *I*. *murucoides* exerts an allelopathic effect on the epiphyte *T*. *recurvata* seed germination by reducing about 58% [14]. We found that the dichloromethane extracts obtained from the eight treatments always inhibited the seed germination of this epiphyte. However, the highest percentages of inhibition occurred when extracts obtained from excluded branches were applied, reaching 60% inhibition (Table 3). In the study of Valencia-Díaz and collaborators [14], the extracts were obtained from the bark of branches that had not been subjected to any treatment of exclusion or damage. If we compare exclusively the percentages of inhibition (30.41%; Table 3) produced with extracts from branches with similar conditions (non-excluded branches, not intentionally damaged and with or without epiphytes) to the percentage of inhibition produced by the dichloromethane extract of *I*. *murucoides* reported in the previous study, we find a significant lower inhibition (Mann-Whitney; *U* = 7, *P* = 0.002). While the extracts obtained are from individuals of *I*. *murucoides* from the same study zone, it is probable that the differences found are due to the temporal variability of the climate, to the availability of resources that affect the composition and concentration of secondary metabolites [1] in branches, or to intrinsic differences (v. gr. seed viability) in the sets of *T*. *recurvata* seeds from different years.

As stated previously, herbivory induces the synthesis of allelochemicals that can perform multiple biological functions [6, 7] and then mediate multispecies interactions. Specifically, in an epiphytic system, xylophagy allows the liberation of defense chemical compounds [38] that may indirectly influence the epiphytes that the host may support. At the other hand, since xylophages can often be found in weakened or diseased tissues in the host [39, 40], epiphytic plants weaken the host and provoke the arrival of xylophages. We found that the Cerambicidae represented the most numerous xylophagous group, but did not find direct evidence that epiphytes or intentional damage to the branches would provoke the arrival of xylophages to the host since, statistically, all of the branches had the same number of xylophages, the same percentage of non-intentional damage and the same epiphyte cover. It is important to mention that our results are based on the data obtained at the end of the experiment, since in order to determine the initial percentage of non-intentional damage and number of xylophages in the branches, it is necessary to destroy the branches prior the execution of the field experiment. However in order to have a reference, we found statistical differences (Mann-Whitney; *U* = 6.00, *P* = 0.018) between the weighted means of a) xylophages/length (cm) of branches of this study (1.75 ± 0.56 xilophages/cm) and b) data of Valencia-Díaz et al. [18] (0.006 ± 0.002 xilophages/cm), where branches were not subject to any treatment (exclusion, damage or epiphyes). This may suggest that xylophages are more abundant in branches subject to any of the treatments performed here. Although both studies were performed in the same population of *I*. *murucoides*, this comparison must be considered with caution because of the temporal variability of xylophages populations.

We did not find evidence that extracts obtained from intentionally damaged branches provoked a greater allelopathic effect in *T*. *recurvata* seed germination, but according to the PCA chemical content analysis, those extracts with intentional damage contain more *trans*-9-hexadecen-1-ol, *cis*-9-eicosen-1-ol and 1-hexadecanol; of these it has been shown that 1-hexadecanol may increase their concentration due to plant injury [41]. When herbivores feed they liberate specific enzymes that activate the host defense mechanisms [1, 2, 42, 43, 44]. The fact that there is no direct relationship between the damage and increased inhibition of germination in *T*. *recurvata* does not preclude the activation of direct defenses against the herbivore. It is possible that the chemicals constituents of the branches that suffered damage can have a direct detrimental effect on the xylophages, but confirmation of this would require studying the insecticidal activity of extracts of *I*. *murucoides* on this guild.

Mesh bag exclusion was done in order to prevent subsequent arrival of xylophages to the experimental branches, but contrary to expectation, the extracts from excluded branches had a greater inhibitory effect on the germination of *T*. *recurvata* seeds. Two of the five chemical compounds correlated positively with the inhibition of seed germination of *T*. *recurvata* were hexadecanoic acid (palmitic acid) and squalene. Palmitic acid has been reported as a constituent of *I*. *murucoides* and as an inhibitor of seed germination of *T*. *recurvata*, also it has been confirmed that commercial compound inhibited 65% the seed germination of this epiphyte [14]. This compound also exerts allelopathic negative effects in parasitic weeds [45]. In the case of squalene, it has been reported that herbivory increases its content in leaves of *Sebastiana adenophora* (Euphorbiaceae) [46], and may act as a sinomone attracting herbivore enemies [47]. It is possible that the weight or the shadow created by the mesh used for exclusion treatments increase the squalene and palmitic acid content.

On the other hand, the treatment of presence/absence of epiphytes did not show differences in terms of the inhibitory effect of the extracts obtained from these two treatments. It is reasonable to consider that the branches with epiphytes would be more shaded than those without epiphytes and thus the chemical constitution could change as occurred in the exclusion treatment; however, it is possible that the coverage provided by the four epiphytes that were transplanted on the branches of this treatment was insufficient to bring about a change in the chemical content of the bark of these branches; in fact PC1 separated control branches along with branches with only epiphytes.

The only interaction of the treatments in which there were differences in the inhibition of seed germination in *T*. *recurvata* was the interaction between exclusion and presence of epiphytes. However, as can be seen from the results, this was determined basically by the treatment of exclusion; i.e., regardless of the presence of *T*. *recurvata*, the greatest inhibition of germination was produced when exclusion of the branches was included in the interaction. In fact, no interaction presented among the eight treatments of this experimental design exercised an inhibitory effect that was distinctively greater than the others. The fact that no statistical differences were found in the inhibition of the germination of *T*. *recurvata* seeds as a function of the treatments does not mean that there may not have been inhibitory effects, but rather that there is no relationship between this effect and the damage while there is a relationship with the exclusion and presence of epiphytes. As stated previously, all of the treatments inhibited germination, a result that corroborates the allelopathic effect of *I*. *murucoides* [14, 27].

At the interspecific level, there is evidence that in host species with a low abundance of epiphytes (*Ipomoea murucoides*, *I*. *pauciflora* and *Bursera fagaroides*) there is a negative association between the xylophages (damage and abundance) and the epiphytes (coverage and abundance) they host. This pattern is most evident in *I*. *murucoides* [18]. We found no evidence to suggest that this multispecies association is mediated chemically; however, we did find evidence that showed a different chemical content in extracts obtained from excluded branches and this may be responsible for the increment of allelopatic activity. If the mesh of excluded branches would seem as a high load of epiphytes, then it is possible that the shade or load generated by epiphytes change also the chemical content and consequently causes more inhibition of the germination of seeds of *T*. *recurvata*.
